# A systematic review of global Q fever outbreaks

**DOI:** 10.1016/j.onehlt.2023.100667

**Published:** 2023-12-27

**Authors:** Tabita Tan, Jane Heller, Simon Firestone, Mark Stevenson, Anke Wiethoelter

**Affiliations:** a Gulbali Institute, School of Agricultural, Environmental and Veterinary Sciences, Charles Sturt University, Wagga Wagga, NSW 2678, Australia; b Melbourne Veterinary School, Faculty of Science, The University of Melbourne, Parkville, VIC 3010, Australia

**Keywords:** *Coxiella burnetii*, Epidemic, Outbreak investigation, Evaluative review

## Abstract

Q fever is an important zoonotic disease with a worldwide distribution. Outbreaks of Q fever are unpredictable and can affect many people, resulting in a significant burden on public health. The epidemiology of the disease is complex and substantial efforts are required to understand and control Q fever outbreaks. The purpose of this study was to systematically review previous investigations of outbreaks and summarise important epidemiological features. This will improve knowledge of the factors driving the occurrence of Q fever outbreaks and assist decision makers in implementing mitigation strategies. A search of four electronic databases identified 94 eligible articles published in English between 1990 and 2022 that related to 81 unique human Q fever outbreaks. Outbreaks were reported across 27 countries and mostly in industrialised nations. Documented Q fever outbreaks varied in size (2 to 4107 cases) and duration (4 to 1722 days). Most outbreaks (43/81) occurred in communities outside of traditional at-risk occupational settings and were frequently associated with living in proximity to livestock holdings (21/43). Indirect transmission via environmental contamination, windborne spread or fomites was the most common route of infection, particularly for large community outbreaks. Exposure to ruminants and/or their products were confirmed as the principal risk factors for infection, with sheep (28/81) as the most common source followed by goats (12/81) and cattle (7/81). Cooperation and data sharing between human and animal health authorities is valuable for outbreak investigation and control using public health and veterinary measures, but this multisectoral approach was seldom applied (14/81). Increased awareness of Q fever among health professionals and the public may facilitate the early detection of emerging outbreaks that are due to non-occupational, environmental exposures in the community.

## Highlights

• Epidemiological features of 81 human Q fever outbreaks were analysed.
• Globally, most large Q fever outbreaks occurred in urban communities.
• Indirect rather than direct contact was the most frequent mode of transmission.
• Nevertheless, proximity to livestock was a common risk factor for Q fever infection.
• Value of inter-agency collaboration during outbreaks was noted, but seldom applied.

## 1 Introduction

Q fever is an important zoonotic disease caused by the bacterium *Coxiella burnetii* [[1], [2], [3], [4]]. Domestic ruminants, in particular cattle, sheep and goats, are the most frequent sources of human infection and Q fever is commonly perceived as an occupational hazard of persons working with livestock [1,5]. However, *C. burnetii* has an extensive range of natural hosts and other animals including dogs, cats, wildlife, rodents and arthropods have been implicated as occasional sources of human infection [[6], [7], [8], [9], [10]]. Inhalation is the main route of human acquisition and typically occurs by close contact with infected animals or airborne dissemination of contaminated dust and aerosols [11,12]. Because the organism remains viable outside of its eukaryotic host for extended periods of time, infection can also occur from contaminated environments or materials [13,14].

Q fever can pose a substantial disease burden and surveillance is conducted in many countries through case notifications from national health systems [15]. While most human infections result in asymptomatic seroconversion, clinical disease can be severe and life threatening, requiring hospitalisation and extensive treatment [[16], [17], [18]]. Diagnosis is challenging because symptoms are non-specific [1,14,19] and complicated laboratory testing is necessary to confirm suspicions of infection [14].

Q fever has been reported across the world, but most outbreaks tend to be sporadic and transient, affecting limited numbers of people. However, outbreaks with sustained and widespread transmission are known to occur and cause significant public health problems. The largest recorded Q fever epidemic was in The Netherlands and resulted in over 4000 notified human cases, lasting from 2007 to 2010. Around 20% of notified cases required hospitalisation for acute disease with even greater numbers of chronic Q fever occurring among exposed groups [20]. Outbreaks require substantial efforts to investigate and control because the epidemiology of Q fever is incompletely characterised. In particular, the variable prevalence in multiple natural hosts and their varying competency for shedding, persistence in the environment, distribution in fomites and capacity for airborne spread. Difficulties encountered in correctly identifying the source of infection and tracing transmission pathways, can lead to broad mitigation strategies that are not well targeted.

Therefore, the purpose of this study was to systematically review and document important epidemiological features, contributing factors and responses from investigations of human Q fever outbreaks. Identifying, evaluating, and summarising the findings of relevant investigations will enhance our understanding of the drivers of outbreaks and aid decision makers in the development of evidence-based prevention and control strategies.

## 2 Methods

This study was registered with the International Prospective Register of Systematic Reviews ([CRD42021238984] PROSPERO, www.crd.york.ac.uk). A comprehensive search strategy was applied to four electronic databases: Web of Science (all databases), CAB Abstracts, Scopus and MEDLINE (Supplementary material S1). Search terms included ‘outbreak’, ‘epidemic’, ‘cluster’, ‘Q fever’ and ‘*Coxiella burnetii*’ and limited to articles published between 1990 and 2023. References were exported into Covidence systematic review software [21]. Following the removal of duplicates, articles were initially screened on title and abstract by the first author (TT). Articles were excluded if they did not describe an epidemiological investigation of a human Q fever outbreak event per se such as articles focussed only on clinical aspects of the disease, seroprevalence, diagnostic techniques or genomic characterisations. For this study, an outbreak was defined as ‘the occurrence of more cases of disease than expected in an area or a specified group over a particular period of time’ [22]. Outbreaks involving multiple pathogens were also excluded to avoid confusion with features of other contributing diseases. Primary journal articles, short reports and letters to the editor were included but books or book chapters, conference abstracts or proceedings, theses and dissertations were excluded. Remaining articles were reviewed on full text by TT. Those outbreaks that were in animals only, were not in English or did not have available full text were excluded. Any ambiguous articles were independently evaluated by two reviewers (AW and JH) and discussed among the research team before a decision was made.

A data extraction sheet was prepared and pilot-tested before being reviewed and finalised by TT and AW. Variables were extracted by TT and included year of outbreak, country, setting, size, duration, type of study, outbreak detection, verification of diagnosis, source, acquisition, transmission, communication between human and animal health, case finding, case definition, descriptive epidemiology, key risk factors, further testing, hypothesis, strength of evidence supporting the hypothesis and prevention/control measures. As part of the data extraction process, each article was evaluated against the recommended epidemiological steps of an outbreak investigation [22]. In addition, the evidence presented in each article was assessed to determine whether it supported the hypothesised outbreak source. This assessment considered whether the evidence was circumstantial or derived from risk factor analysis and/or additional sampling (Supplementary material S3). The unit of interest was defined as an outbreak of Q fever. Where an article reported on more than one outbreak, data were extracted for each outbreak onto a new line. If more than one article reported on the same outbreak, data were first individually extracted for each article and then summarised onto one line as a single outbreak.

Descriptive analysis was performed on variables collected as numerical and categorical data. For variables extracted as free text, data were examined for themes before being categorised and descriptively analysed. Datawrapper [23] was used to create a chloropleth map where the number of outbreaks per country is represented by the colouring of the country. Areas with higher numbers of outbreaks are represented by darker colours, while lower numbers of outbreaks are represented by lighter colours. Outbreak size was categorised as small, medium and large using tertiles of the data range from the number of cases. Linear regression analysis for outbreak size and duration, and graphs were undertaken using the R Statistical Package version 3.4.4 [24]. A narrative synthesis was used to summarise and explain the findings because the range of research designs producing qualitative and quantitative findings did not allow for a statistical synthesis, such as a meta-analysis [25]. This study is reported following the ‘Preferred Reporting Items for Systematic Reviews and Meta-Analyses’ (PRISMA) checklist to ensure clarity [26] (Supplementary material S2).

## 3 Results

### 3.1 General characteristics

**Fig. 1:**
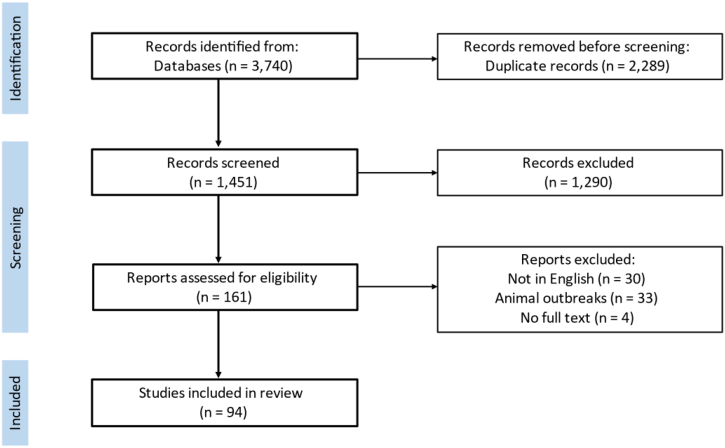
Flow diagram for the article selection process.

From the initial search across four databases, 3740 articles were identified of which 2289 duplicates were removed. Screening using predefined inclusion and exclusion criteria removed a further 1451 articles, leaving 94 articles for data extraction (Fig. 1.).

The 94 eligible articles represented 81 unique outbreaks (Supplementary material S3). Twelve articles that were overviews of the extensive outbreak in the Netherlands [5,[27], [28], [29], [30], [31], [32], [33], [34], [35], [36], [37]] were summarised into one outbreak. Six research articles investigating clusters within the larger Netherlands outbreak were considered as individual outbreaks because they featured an isolated source affecting discrete human populations in demarcated locations [34,[38], [39], [40], [41], [42]].

**Fig. 2:**
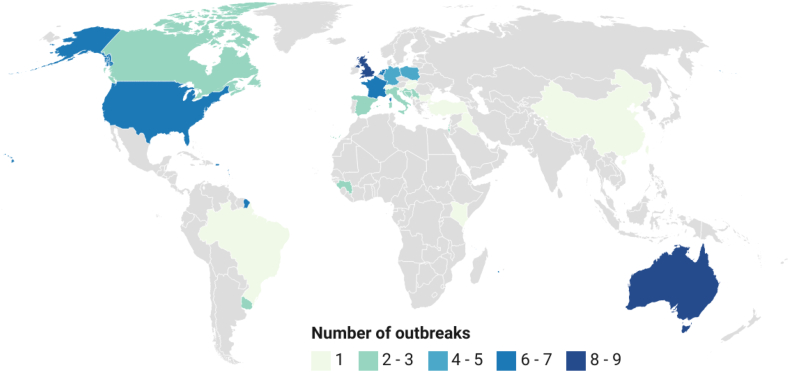
Chloropleth map of Q fever outbreaks reported globally that were included in a systematic review of global Q fever outbreaks.

Outbreaks were described across 28 countries with the highest number reported in Australia (*n* = 9), UK (*n* = 8), France and The Netherlands (each *n* = 7) and the USA (*n* = 6) (Fig. 2). The median number of cases identified per outbreak was 28 (range: 2 to 4107). Sixty-five outbreaks were small comprising ≤80 cases, 8 were medium, and 10 were large with over 160 cases. The median outbreak duration was 76 days (range: 4 to 1722). After accounting for the largest Netherlands outbreak as an outlier because it exhibited extreme values that masked patterns from the rest of the data, it was found that increasing magnitude and duration were not always associated (*p* = 0.26). This indicates that other factors, such as variable persistence of infection source and methods of propagation, can influence the size of the outbreak.

### 3.2 Detection and confirmation of outbreaks

Factors that triggered recognition of an outbreak were reported for 67 outbreaks and included an increased number of people presenting with fever, atypical pneumonia or requiring hospitalisation who were clustered within an organisation or geographical location (*n* = 28 outbreaks); an increase in the number of diagnoses notified to authorities by mandatory laboratory reporting from a local area (*n* = 23); follow-up patient interviews that revealed family members or co-workers with similar symptoms (*n* = 7); or systematic testing of a population for Q fever (n = 2). Initial detection of *Coxiella burnetii* in livestock resulted in the identification of seven human outbreaks [[42], [43], [44]]. This was facilitated by mandatory veterinary notification of livestock infections [42,44]. Laboratory testing (serology and polymerase chain reaction) was used in all investigations to confirm a diagnosis of Q fever infection in humans.

Methods for case finding included soliciting directly from a list of exposed individuals; public alerts; identification by health care providers; review of medical records or laboratory samples; and monitoring national statutory health surveillance system for cases. Case finding was usually accompanied by the collection and testing of blood samples to definitively identify infected individuals. Demographic information, clinical data and distribution of exposure variables were collected from human cases, and sometimes non-cases depending on the objective of the study. These were mainly acquired through questionnaires, interviews and sometimes from medical or laboratory records. Most outbreak investigations were descriptive (*n* = 45) but others were reported in the form of case-control studies (*n* = 24), retrospective cohort studies (*n* = 8), cross-sectional studies (*n* = 3) and a case-case study (*n* = 1).

Further investigation to confirm the proposed hypothesis (animal source and method of transmission) detailed the collection of animal and environmental samples as well as meteorological and animal-level data. Animal samples (*n* = 53) included blood, milk, faeces, vaginal swabs, rectal swabs and aborted material. Environmental sampling (*n* = 20) included dust, aerosols and fomites such as soil, straw and animal bedding. Meteorological data (n = 20) included temperature, rainfall and prevailing winds to assess potential for windborne spread from the animal source. Animal-level data (*n* = 15) were collected on the location of animals surrounding the outbreak area and about animal health, breeding and abortion history.

Communication between human and animal health organisations was described in less than half (35/81) of outbreak investigations. Where communication occurred, local public health authorities usually took the lead as principal investigators with veterinary services in a minor adjunct role. Veterinary services provided intelligence on possible animal sources such as location and proximity of farms to human dwellings and animal health data on breeding history and abortions. They also aided in the collection and testing of animal samples. Collaboration across sectors for outbreak response was described for 14 outbreaks. Out of these, 6 outbreak responses established a multi-disciplinary team from several human and animal health organisations, which is the highest level of cooperation [40,[45], [46], [47], [48], [49]]. In some countries (e.g. Netherlands, Poland, Serbia) data sharing was supported by a legal framework and source identification and control was based on cooperation between human and veterinary health services [34,39,42,44,48,50].

### 3.3 Infection source, acquisition and transmission routes

**Fig. 3:**
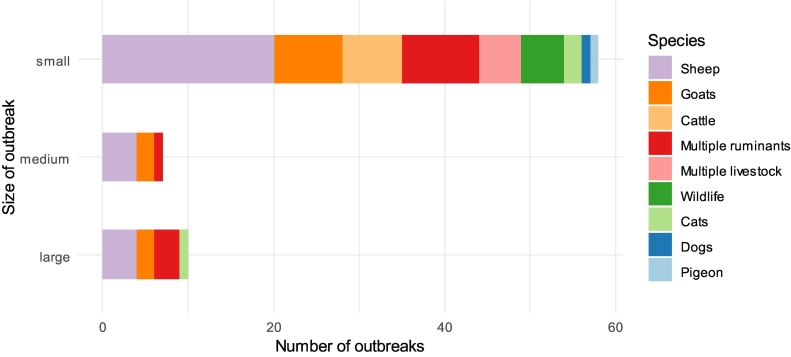
Species as a source of infection for outbreak size. Outbreak size is specified by tertiles of the data range collected (small: 1–80, medium: 81–160, large: >160).

Overall, 15 animal species were implicated as sources of Q fever outbreaks. Sheep (*n* = 28) were the most common followed by goats (*n* = 12) and cattle (*n* = 7). In 13 outbreaks, multiple ruminants (combinations of cattle, sheep and goats) were potential sources of infection. Multiple livestock species were suspected in five outbreaks and included pigs [51], poultry [52] and camels [53]. Cats (*n* = 3), dogs (*n* = 1) and pigeons (n = 1) were linked to a small number of outbreaks in humans. Wildlife was associated with five outbreaks and included the three-toed sloth (*Bradypus tridactylus*) [54], Formosa barking deer (*Muntiacus reevesi*) [55], field deer (*Ozotoceros bezoarticus*) [56], capybara (*Hydrochoerus hydrochaeris*) [57], kangaroos (Macropodidae), feral animals and ticks [10]. No source was identified in six outbreaks. Parturient or abortion events were a contributing factor for many outbreaks (40/81), particularly for domestic species. A broader range of animals were identified as sources for small sized outbreaks, whereas larger outbreaks were mostly attributed to ruminant species, especially sheep (Fig. 3). Evidence provided by articles to implicate the animal source was variable and ranged from the incidental presence of a possible source near the human outbreak through to statistical analyses of animal associated risk factors with laboratory testing of animal and environmental samples for the pathogen.

Acquisition of infection in humans was classed as traditional occupational (*n* = 17), non-traditional occupational (*n* = 20) and community (*n* = 43). Occupations considered traditionally at risk are those associated with the livestock industry such as veterinarians, abattoir workers and farmers. Non-traditional occupations do not routinely involve work with livestock or their products and are not typically considered at risk. Community acquisition refers to infection of people in the wider population outside of workplace settings. The list of settings for each type of acquisition is detailed in Table 1.

**Table 1:** Specific settings for traditional occupational, non-traditional occupational and community acquisition and the number of outbreaks reported for each setting.

**Setting**	**No. of outbreaks**⁎
**Community**	**43**
Residential areas (cities, towns, villages)	26
Facilities (e.g. prison, psychiatric care, drug rehabilitation)	9
Tourism/visitation (safari, petting zoo, farm)	4
Schools	2
Households (due to pets whelping at home)	2
Raw milk drinkers	1
**Non-traditional occupational**	**20**
Manufacturing and service industries • cardboard, cosmetics, hoists and chains • hospital kitchen, waste sorting, pet courier	7
Military/police force	6
Research/laboratory	4
Non-ruminant farm (pigeon, horse ranch)	2
Animal refuge	1
**Traditional occupational**	**17**
Ruminant farms	7
Abattoir	7
Agricultural/Veterinary education	2
Saleyard	1

⁎ Many outbreaks had more than one mode of acquisition but were classed according to the predominant group affected so that the categories were mutually exclusive. The setting was unknown for one outbreak.

**Fig. 4:**
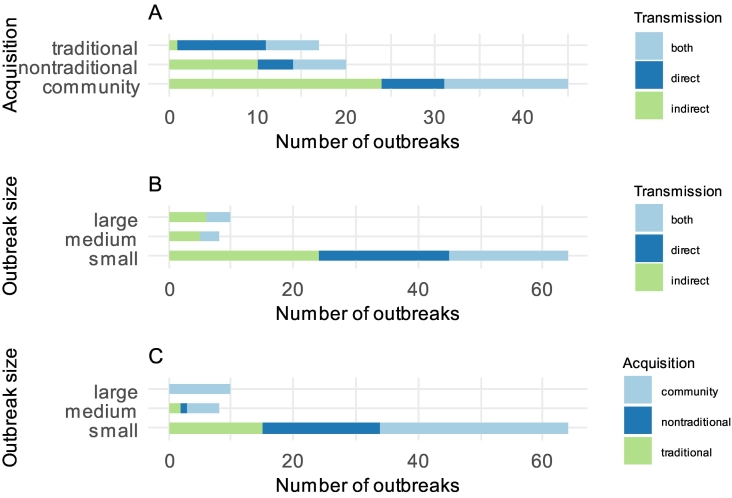
(A) Route of transmission for each type of acquisition. (B) Route of transmission for outbreak size. (C) Type of acquisition for outbreak size. Outbreak size is specified by tertiles of the data range collected (small: 1–80, medium: 81–160, large: >160).

Transmission of disease was categorised as direct (*n* = 21), indirect (*n* = 36) or both (*n* = 24). Direct transmission requires close contact with animals, their products or waste. Indirect transmission was via environmental contamination, windborne spread or on fomites. Indirect transmission was the most common route of infection for community acquisition and non-traditional occupational acquisition, whereas direct transmission was more likely for traditional occupational acquisition (Fig. 4A). Windborne spread from the animal source some distance away from human populations was implicated in 19 outbreaks. Transmission via fomites such as dust, strawboards [58] and on the garments or footwear of animal attendants [45,59,60] were reported in four outbreaks. Unusual examples of direct transmission include ingestion of raw milk [44,61,62] and live cell therapy in which foetal sheep cells were injected intramuscularly into human recipients [63].

Indirect transmission was the most likely route for medium and large Q fever outbreaks but both direct and indirect transmission was important for small outbreaks (Fig. 4B). Large and medium sized outbreaks were more likely to be community rather than occupationally acquired, whereas small outbreaks were more evenly distributed between community, non-traditional occupational and traditional occupational acquisition (Fig. 4C).

### 3.4 Risk factors for infection

Statistical information was provided on the measure of association (odds ratio/risk ratio/relative risk/prevalence ratio; confidence intervals/*p*-values) between suspected risk factors and infection/disease status for 48 outbreaks. Exposure to ruminants and/or ruminant products was the most commonly reported risk factor (*n* = 31). Proximity to livestock facilities was also an important risk for infection or disease (*n* = 22) and increasing distance from the likely source, usually an infected farm, was associated with a gradient of decreasing infection (*n* = 7). A complete list of risk factors investigated is provided in the Supplementary material S4.

### 3.5 Control and prevention

Control and prevention measures were reported for 64 outbreaks, ranging from no action to severe restrictions with widescale animal destruction (Supplementary material S5). Hygiene practices and biosecurity (*n* = 23) were the most utilised veterinary or environmental interventions, which involved ceasing high risk practices, appropriate disposal of animal waste and disinfection of contaminated premises, facilities or transport routes. Reducing human contact with the source (*n* = 13) and education and raising awareness among the community, farmers and health care providers (*n* = 11) were the most common interventions applied to improve human understanding and behaviour towards the risks of Q fever. The effectiveness of control efforts was seldom reported. In some outbreaks, cases continued to appear despite actions to remove the animal source [64]. Specific issues identified from investigations in this review that hindered disease control include: dispersal through fomites and animal matter that serve as a hidden source of infection [[58], [59], [60]], capacity for distant windborne spread [39,65], low infectious dose [66], multiple natural hosts [10], presence of sylvatic cycle transmission [57] and an absence of validated measures for environmental contamination [34,67].

## 4 Discussion

This study identifies new global evidence regarding the prominence of large outbreaks in urban community settings and the important role of indirect transmission in the spread of Q fever disease. More than two-thirds of the outbreaks worldwide occurred in communities outside of traditional at-risk settings, of which many individuals had no contact with livestock. In this review, indirect contact was found to be the most frequent mode of transmission and living in proximity to livestock was a common risk factor for infection. This is in stark contrast to historical beliefs that Q fever is primarily an occupational hazard of individuals working directly with livestock (farmers, veterinarians, livestock transporters, abattoir workers, shearers and wool classers) or that it is only limited to rural areas [14,66,[68], [69], [70]].

Q fever outbreaks can be difficult to identify because most seroconversion in humans is asymptomatic, and outbreak detection involves several patients with illness severe enough to present for medical care. Health professionals need sufficient knowledge to recognise the disease and navigate the complicated diagnostic process to confirm infection [5,71] and several articles observed the requirement of an astute individual to discern a link between cases [9,10,45,50]. Outbreak detection is particularly problematic in urban residents for whom *Coxiella burnetii* would be considered an uncommon zoonosis due to their lack of conventional risk factors of direct exposure to livestock [10,14,69,[72], [73], [74], [75]]. Consequently, it is highly likely that the incidence and extent of infection is underestimated. Issues with underdiagnosis or misdiagnosis in settings with no occupationally associated risk can lead to a delay in outbreak investigations, hinder tracing of the source and compromise the effectiveness of mitigation measures [74,76]. Because lack of disease familiarity is a known barrier to Q fever diagnosis, education to improve awareness and the breadth of risk factors for infection among health professionals and the public may facilitate the early detection of future Q fever outbreaks that are due to non-occupational, environmental exposures in the community [5,14,48,69,76].

A range of animals were documented in this review as being sources of infection with ruminant livestock, particularly sheep, asserted as the predominant source. This is in line with previous publications [1,5,77,78]. However, it is possible that the common perception that sheep are frequently the source of infection, their relative abundance in countries reporting outbreaks and comparatively large herd sizes may have created a bias for implicating this species. The contribution of goats as a source of infection may have increased if outbreaks were reported from other countries, such as central Africa, where goats occur in greater numbers [79]. Furthermore, evidence provided by several investigations for linking the outbreak with the presence of sheep was circumstantial rather than substantiated by risk factor analyses or positive animal samples [67,80,81]. The variable extent and quality of reported investigations underscore inconsistencies and limitations in the approach to Q fever surveillance, emphasizing the need for a more comprehensive and standardized methodology. Other domestic mammals, poultry and wildlife appear to have a minor role in Q fever outbreaks but knowledge around the effectiveness of non-ruminant species in maintaining and transmitting infection to humans is incomplete [14,77]. With the growing spectrum of animals discovered to be reservoirs, species attribution requires further exploration and investigators should be wary of ruling out alternative sources of Q fever outbreaks.

There was no consistent pattern for the Q fever outbreaks reviewed as some were explosive with hundreds of people affected in a short space of time, whereas others were protracted with several cases over many months. The nature of outbreaks could not be predicated on the presence of individual factors commonly accepted as having a high infectious potential. For example, increasing case numbers were not always associated with greater animal density or intensive livestock rearing, even though these systems provide the ideal environment for the multiplication and spread of *C. burnetii* [45,82]. An infected intensive dairy goat farm raising thousands of animals resulted in less than two dozen human cases over the course of two years [45] whereas a single lambing ewe was responsible for hundreds of human cases in less than two months [47]. The variable epidemic patterns of outbreaks reported in this review emphasizes that Q fever epidemiology is the product of multiple factors that influence the dynamic interactions between hosts, animal reservoirs and the environment [5,77,83,84]. Several factors such as human density, patterns of land use, human behaviour, host immunity, timing of parturition and meteorological conditions have been found in this review and other studies to contribute to largescale outbreaks and should be considered in addition to other putative risk factors [5,77,83,84]. However, the unpredictable nature of many of these factors, along with the likely contribution of seldom studied epidemiological parameters, such as duration of infectivity in the environment or efficiency of non-ruminant hosts to transmit infection, is likely to confound our understanding of Q fever outbreaks.

The purpose of outbreak investigations is to eliminate the source and prevent new cases from occurring [85,86]. In many outbreaks, humans inadvertently act as sentinels for *Coxiella burnetii*, as identification of human cases precedes realization that the pathogen is present. This leads to time delays in response efforts and challenges around backwards tracing to determine the source. Failure to definitively identify the source and interventions that focus only on preventing direct transmission while neglecting indirect transmission pathways can limit the effectiveness of control efforts. Considering the magnitude of animal and human involvement and widespread environmental dissemination, a One Health approach that provides a holistic framework for integration across multiple sectors is necessary for an outbreak response to be successful [15,87]. Cooperation between disciplines is valuable not just for early detection and identifying potential sources of infection but also for enhancing disease control through a combination of public health, veterinary and environmental measures. The value of collaboration to improve health outcomes and reduce the impact of an outbreak with timely detection and control, was noted in several studies [44,45,48,64,[88], [89], [90], [91], [92]]. Notwithstanding this, multidisciplinary collaborations across agencies occurred infrequently with investigation efforts mainly the responsibility of human health authorities. Barriers to interdisciplinary collaboration have been explored in other studies and include lack of communication, disputes between stakeholders, inadequate policies and pathways for coordination, absence of a vision for One Health applications, legislative challenges, and misunderstandings of zoonotic disease epidemiology [15,87,93,94]. Disease outbreaks can serve as an opportunity to overcome barriers and foster the integration of One Health [94] because of the urgency for action and the common goal of health protection. Some outbreak responses in this review have led to improved communication between physicians, public health authorities, veterinarians and farmers in the affected area [64], sharing of epidemiological and medical information [88], integrated human-animal surveillance [89], mandatory veterinary notification of livestock cases to assist identification of human infection [5,34] and development of multi-disciplinary teams for comprehensive risk evaluation and consensus control recommendations [45]. There are also economic benefits of sharing resources with improved and timely detection and control [87]. When government authorities appreciate the benefits of multisectoral collaboration for outbreak response, the establishment of a legislative framework can facilitate cooperation between human, veterinary and environmental health services [34,39,42,44,48,50].

Although this review aimed to capture all relevant published literature about Q fever outbreaks, there were several limitations. Only articles published in English with available full-text were included and outbreaks reported from countries in other languages are not reflected in the results. Most outbreaks were reported from the industrialised nations of western Europe, North America and Australia with a very small number from articles from Asia, Africa and Latin America. This spatial bias likely reflects the preference for publications from English-speaking countries and higher levels of funding for surveillance in developed countries compared to developing nations. It was surprising that no Q fever outbreaks were reported from other developed countries such as Japan or in the Middle East. This could be due to a perception that Q fever is not a problem in those countries, rather than the inability for detection. *Coxiella burnetii* is known to have a worldwide distribution [68,95] and the uneven geographical distribution of reported outbreaks are likely due to insufficient interest, reduced capacity for detection and diminished likelihood for publication in English rather than a true absence of disease. Not all outbreaks worldwide have been included in this review and the findings may not be representative of every setting. However, the deficiencies in managing Q fever outbreaks encountered by developed countries with adequate resources are likely to be universally applicable. These result in suboptimal outbreak responses and include inability to always identify a source, control measures used that are not broadly encompassing, limited knowledge around Q fever in non-traditional settings, and infrequent application of a One Health approach.

## 5 Conclusion

Many factors influence Q fever outbreaks which can manifest in a variety of settings and with differing epidemic patterns. Q fever is commonly perceived as a hazard of persons working with livestock, but an absence of animal contact does not preclude infection due to the marked capacity for indirect transmission, leading to the likely underestimation of risk for outbreaks in urban communities. Lack of suspicion for Q fever in these populations can lead to difficulties in outbreak recognition and consequently delay a response. Challenges to outbreak management appear to be broadly applicable. Increasing awareness of the disease and application of a One Health approach would aid early detection and improve response, resulting in economic benefits and better health outcomes. Examples from this review include creating frameworks that support interdisciplinary communication, data sharing and integrated surveillance. Although there are obstacles to joint action by different agencies, outbreaks have been shown to offer a practical opportunity to reduce these barriers and foster collaboration. Demonstrating the advantages of a One Health approach to policy makers may encourage the development of legislation and formal pathways that facilitate collaboration.

## CRediT authorship contribution statement

**Tabita Tan:** Conceptualization, Methodology, Formal analysis, Visualization, Writing – original draft. **Jane Heller:** Supervision, Conceptualization, Methodology, Writing – review & editing. **Simon Firestone:** Conceptualization, Writing – review & editing. **Mark Stevenson:** Conceptualization, Writing – review & editing. **Anke Wiethoelter:** Conceptualization, Methodology, Formal analysis, Supervision, Writing – review & editing.

## Declaration of competing interest

None.

## Data Availability

Articles included in the review and data extracted are available in Supplementary materials.

## Appendix A Supplementary data

Supplementary material 1 S1. Search strategy. Image 1

Supplementary material 2 S2PRISMA checklist. Image 2

Supplementary material 3 S3Data extraction sheet. Image 3

Supplementary material 4 S4 List of exposure risk factors identified. S5 List of control and prevention measures identified. Image 4
