# Supplementary material for: A systematic review of global Q fever outbreaks
Source: One Health. 2023 Dec 27;18:100667. doi: 10.1016/j.onehlt.2023.100667 (PMC11247264; doi:10.1016/j.onehlt.2023.100667)
Supplement: Supplementary file 1 — Supplementary material 1 S1. Search strategy. [file mmc1.pdf]

Supplementary materials S1

Search strategy for the epidemiological investigation of global Q fever outbreaks

Database	Search strategy used	Number of results	Date of search
Web of science	TS = (Q fever OR Coxiella OR Coxiellosis) AND TS = (outbreak OR epidemic OR cluster) Timespan: 1990-01-01 to 2023-02-21	1461	21/2/23
CAB Abstracts	("Q fever" OR "Coxiella" OR "Coxiella burnetii" OR “Coxiellosis”) AND ("outbreaks" OR "epidemics" OR "clustering" OR "clusters") AND yr:[1990 TO 2023]	787	21/2/23
Scopus	(TITLE-ABS-KEY ("Q fever" OR coxiella OR coxiellosis) AND TITLE-ABS-KEY (outbreak OR epidemic OR cluster)) AND PUBYEAR > 1989 AND PUBYEAR < 2024	1020	21/2/23
Medline (Ovid)	(exp Q fever/ OR exp Coxiella burnetii/ OR exp Coxiella/) AND (exp Disease Outbreaks/ or exp Epidemics/)	472	21/2/23
