# Supplementary material for: A systematic review of global Q fever outbreaks
Source: One Health. 2023 Dec 27;18:100667. doi: 10.1016/j.onehlt.2023.100667 (PMC11247264; doi:10.1016/j.onehlt.2023.100667)
Supplement: Supplementary file 4 — Supplementary material 4 S4 List of exposure risk factors identified. S5 List of control and prevention measures identified. [file mmc4.pdf]

**Supplementary materials S4**

| **Exposure risk factors** | **No. of outbreaks** |
|---|---|
| Exposure to ruminants and/or ruminant products | 31 |
| Specific to an occupation or institutional setting (e.g. abattoir, factory, school) | 14 |
| Location or residence in proximity to livestock | 22 |
| Exposure to other animals (e.g. dogs, cats, pigeons, poultry, wildlife) | 10 |
| Not using PPE (e.g. mask) or office HEPA filter | 4 |
| Cleaning and aerosol generating activities (e.g. high pressure cleaning, grass mowing, brush cutting) | 3 |

**Supplementary materials S5**

| **Control and prevention measures** | **No. outbreaks** |
|---|---|
| ***Veterinary and environmental interventions*** | |
| Hygiene practices and biosecurity on farm/facility | 23 |
| Destruction or relocation of animals | 11 |
| Vaccination or treatment of livestock with antibiotics | 11 |
| Movement restrictions on animals or products | 9 |
| Surveillance for infection in the animal population | 9 |
| Closure or cleaning of facilities or residential areas associated with the outbreak | 9 |
| Improving ventilation systems and air filtration in facility | 2 |
| ***Human immunity and behaviour modification*** | |
| Reducing human contact with source | 13 |
| Education and raising awareness | 11 |
| Vaccination of workers | 9 |
| Training in the use of personal protective equipment such as masks | 6 |

| | |
|---|---|
| Surveillance of at-risk population | 6 |
| Provision of policies and guidelines at workplaces or institutes | 4 |
| Interruption of blood donations | 3 |
